# ‘Big data’ in mental health research: current status and emerging possibilities

**DOI:** 10.1007/s00127-016-1266-8

**Published:** 2016-07-27

**Authors:** Robert Stewart, Katrina Davis

**Affiliations:** Department of Psychological Medicine, Institute of Psychiatry, Psychology and Neuroscience, King’s College London, Box 63, De Crespigny Park, London, SE5 8AF UK

**Keywords:** Big data, Mental disorders, Epidemiology, Electronic health records

## Abstract

**Purpose:**

‘Big data’ are accumulating in a multitude of domains and offer novel opportunities for research. The role of these resources in mental health investigations remains relatively unexplored, although a number of datasets are in use and supporting a range of projects. We sought to review big data resources and their use in mental health research to characterise applications to date and consider directions for innovation in future.

**Methods:**

A narrative review.

**Results:**

Clear disparities were evident in geographic regions covered and in the disorders and interventions receiving most attention.

**Discussion:**

We discuss the strengths and weaknesses of the use of different types of data and the challenges of big data in general. Current research output from big data is still predominantly determined by the information and resources available and there is a need to reverse the situation so that big data platforms are more driven by the needs of clinical services and service users.

## Introduction

‘Big data’ resources for research have attracted increasing interest across healthcare, but applications in mental health have remained relatively limited to date. Big data challenges are traditionally characterised as those of volume (size of datasets), velocity (rapid, ‘real-time’ acquisition of data), and variety (multiple sources and types), with ‘variability’ and ‘veracity’ more recently added to reflect the unreliability of information arising from some sources [1]. There are numerous examples of different sources of big data which might be utilised for health research, including those derived from large biological sample collections, complex investigations (e.g. imaging), devices, and social media. With growing accessibility to large volumes of data accumulating in routine clinical practice following the shift of medical records from paper to electronic formats, clinical notes are also potential big data resources for researchers. There has been a long history of using routine data in mental health research, from the earliest studies of asylum records through the growth of the ‘case register’ in the mid- to late twentieth century. However, larger volumes of information are now accumulating in electronic format than would have been conceivable 20–30 years ago, which potentially transforms data-based investigations. We feel that it is timely to review the information resources being used for big data research, their current scope and coverage, and the nature of the research emerging.

## Method

In a narrative literature review, we sought to ascertain and collate studies where big data approaches had been used in research on mental illness and treatment. Aiming at a representative rather than exhaustive list, the authors used recent reviews [2–10] to extract names of projects to use as key words for a Google Scholar search, adding also the requirement for the terms “mental health” or “psychiat*” for non-specialist sources, restricted to those since 2009. Where no relevant papers were returned, a simple web search for the project was also carried out to check for name changes and non-academic outputs. Forward bibliographic searching was also carried out to identify papers that had cited the reviews above, in an attempt to identify more recent projects. The authors included projects that demonstrated a reach in terms of massed healthcare data, and papers that had used big data approaches for mental health research. Our review was limited to English-language papers, and quantitative and qualitative studies about opinion regarding use of healthcare data were not included. Information was extracted on the name of the project (where given), the database used, the data sources for the database, and the geographical setting. The studies themselves were categorised into disease- or medication-specific, or other topics.

## Results

Data resources identified and their international distribution are summarised in Table 1. In all, we identified 84 examples of databases that had been used to provide big data answers to mental health research questions, of which 24 are specific to mental health and related topics. Geographically, most data resources were found in the United States, with few specific national resources identified outside North America and northern/western Europe. However, there were a number of examples found of multinational and multi-continent collaborative resources, centred mostly on neurodegenerative or neurodevelopmental disorders. What should also be evident from Table 1 is the large number of databases being used for mental health research which are not themselves specific to mental health over and above any other specialty.

**Table 1:** Resources arranged geographically

Region/nation	Database	Mental health specific?	Description	Example publication
Middle East, Asia and Australasia				
Middle East	Clalit Health Services	No	National. Covers 55 % Israeli population	Hammerman et al. [77]
	Israeli Psychiatric Case register	Yes	National. Secondary care psychiatry. Since 1950	Lichtenberg et al. [78]
Far East	Hong Kong Hospital Authority	No	Covers 95 % secondary care in HK	Cheung et al. [79]
	Seoul National University	No	Local secondary care	Park et al. [80]
	Taiwan National Health Insurance Database	No	National. Covers 96 % Taiwan population	Chen et al. [81]
Australia	Mental Health National Outcomes and Casemix	Yes	National. Secondary care psychiatry. Since 2003	Burgess et al. [47]
	Western Australia admin	No	Regional (3.7 m people). Mental health sub-group. Up to 50 years data	Lawrence et al. [82]
Multi-country (Asia)	Pan-Asian SNP Consortium (HUGO)	No	Research database	Ngamphiw et al. [83]
Europe				
Western Europe	Asturias Cumulative Psychiatric Case Register (RACPAS)	Yes	Spain. Regional (1 m people). Secondary care psychiatry	Bobes et al. [84]
	Gmünder ErsatzKasse (GEK)	No	Germany. National. Large health insurer (6 % population, around 5 m people)	Sauer et al. [85]
	German Research Network on Depression/DGPPN-BADO	Yes	BADO is national minimum data set for inpatient psychiatry. Depression network from 10 heterogeneous hospitals	von Wolff et al. [48]
	Health Search Database	No	Italy. National. Primary care data (1.5 % population, around 1 m people)	Sultana et al. [86]
	Marseille/French National Health Insurance Fund	No	Regional. Prescription data	Bocquier et al. [87]
	Regensberg Hospital/DGPPN-BADO	Yes	Germany. Local. BADO is minimum data set from psychiatric inpatients	Frick et al. [88]
	South Verona Community-Based Mental Health Service	Yes	Italy. Local. Secondary care psychiatry. 25 years+ of data	Donisi et al. [89]
	Zurich/Swiss psychiatric case register	Yes	Regional. Secondary care psychiatry. 25 years+ of data	Lay et al. [90]
United Kingdom	Clinical Practice Research Data link (CPRD), formerly General Practice Research Database (GPRD)	No	National sample primary care providers. Some data open access (NIHR.ac.uk)	Margulis et al. [91]
	Clinical Record Interactive Search (CRIS)	Yes	Local secondary care psychiatry. South London and Maudsley Biomedical Research Centre (SLaM BRC) Case Register. 200,000+ people	Perera et al. [20]
	Generation Scotland	No	Regional (Scotland). Research database. Family based cohort	Fernandez-Pujals et al. [92]
	GRiST	Yes	Multiple locations, primary and secondary care psychiatry. Mental health risk assessment software	Buckingham [50]
	Public Health England Mental Health Dementia and Neurology Intelligence Network	Yes	Regional (England). 22 ‘indicators’ from mixed administrative sources	Wilkinson et al. [93]
	QResearch GP database	No	National sample primary care providers. 600 practices, around 12 m people	Coupland et al. [94]
	The Health Improvement Network (THIN)	No	National sample primary care providers. 10 m people, broadly representative of population	Osborn et al. [36]
	UK Biobank	No	National sample 500,000 volunteers. Research database	Smith et al. [58]
	Secure Anonymised Information Linkage (SAIL)	No	Linked data from a range of healthcare sources covering Wales (population 3 m)	John et al. [95]
	PsyCymru	Yes	An e-cohort of around 12,000 psychosis cases in Wales linked to SAIL data	Lloyd et al. [96]
Scandinavia	Danish Psychiatric Central Research Register	Yes	National. Secondary care psychiatry with extensive national linkage	Munk-Jorgensen and Ostergaard [97]
	deCODE Iceland	No	National opt-in commercial/research database	Thorgeirsson et al. [98]
	Dutch National Survey in General Practice	No	National sample primary care providers	Maas et al. [99]
	Finnish Hospital Discharge Register	No	National. Inpatients. Linked to other national registers	Haukka et al. [100]
	Mid-Netherlands Psychiatric Care Register	Yes	Regional—Utrecht and surrounding areas, population 760 k. Secondary care psychiatry	Braam et al. [101]
	Norwegian Patient Register	Yes	National. Secondary care psychiatry. Linked to other national registers	Evensen et al. [102]
	Odense University Pharmaco-epidemiologic Database	No	Denmark. Local prescription database with linkage	Hansen et al. [103]
Eastern Europe	Hungarian National Health Insurance Fund	No	National. Prescription-with-indication database	Katona et al. [104]
Multi-country (Europe)	European Observatory on Health Systems and Policies	No	Health services. Produces country-based reports	Dlouhý and Barták [105]
	European Prevention of Alzheimer’s Dementia (EPAD) project	Yes	A European Innovation Medicines Initiative	Ritchie et al. [106]
	European Autism Interventions	Yes	A European Innovation Medicines Initiative	Murphy and Spooren [107]
	Nordic population-based prescription database	No	Pharmaco-epidemiology using databases from five countries	Zoëga et al. [108]
	PROTECT-EU	No	Pharmaco-vigilence using databases in three countries	Requena et al. [109]
	Refinement	Yes	Mental health services. Population data and service inventory	Sfetcu et al. [56]
America				
Canada	Canadian Chronic Disease Surveillance System (CCDSS)	Yes	National. Will specifically monitor excess mortality in people with psychiatric diagnosis	Lesage et al. [110]
	Canadian Primary Care Sentinel Surveillance Network	No	National sample primary care providers	Wong et al. [35]
	OntarioMD	No	Regional, primary care providers	Hwang et al. [111]
	Ontario Mental Health Reporting System	Yes	Regional, based on interRAI MH dataset for psychiatric inpatients	Perlman et al. [112]
	Saskatchewan Health Databases	No	Regional, multisource. 25 years+ of data	Meng et al. [113]
USA	23andMe	No	National. Commercial genotyping database, self-report	Tung et al. [114]
	Agency for Healthcare Research and Quality (AHRQ) Healthcare Cost and Utilisation Project (HCUP)	No	National sample hospital care providers. Databases and software through Federal-State-Industry partnership	Smith et al. [115]
	Alzheimer’s Disease Genetic Consortium	Yes	Distributed network of sample of healthcare providers	McDavid et al. [116]
	CDC data surveillance systems, including national ambulatory care survey	No	National. A number of monitoring systems and surveys	Olfson et al. [117]
	Data QUEST	No	Sample of 15 primary care providers in five states	Estiri et al. [118]
	Electronic medical records and genomics network (eMERGE)	No	Distributed network of five leading academic medical centres for biobanking, includes Alzheimer’s cohorts	Kho et al. [40]
	Group Health Research Institute (GHRI)	No	Healthcare management organization (HMO). HMO network member	Lin et al. [119]
	Health Plan Employer Data and Information Set (HEDIS)	No	National. Set of performance measures used by most health plans in USA. Managed by National Committee for Quality Assurance (NCQA)	Clark et al. [120]
	Informatics for integrating biology and the bedside (i2b2)	No	Local secondary care. Biobank affiliated with Harvard Medical Schools	Perlis et al. [26]
	U.S. Food and Drug Administration (FDA) Mini-Sentinel, including Innovation in Medical Evidence Development and Surveillance (IMEDS)	No	National (currently sample) medication-based database, aiming to create active monitoring system	Raebel et al. [121]
	Kaiser Permanente, including KP Research Program on Genes, Environment and Health (RGEH)	No	Regional sample. HMO based in Northern California, 3.4 m insured	Young et al. [122]
	Mayo Clinic	No	Local secondary care provider. Based in Minnesota, also contributes to Olmsted County/Rochester projects	Sohn et al. [123]
	MarketScan Research Database	No	National sample. Commercial claims and encounters database from mix of providers	Watkins et al. [124]
	Medicaid & Medicare data	No	National sample. Government reimbursed healthcare activity. Data accessed through CMS.gov or a variety of platforms, including MarketScan and HEDIS	Medicaid Medical Directors Learning Network [125]
	Mental Health Research Network at Health Care Systems Research Network, formerly HMO research network	No	National sample. Distributed network of up to 17 HMOs with virtual data warehouse. Potentially 11 m population in 11 states	Ahmedani et al. [126]
	Multiparameter Intelligent Monitoring in Intensive Care (MIMIC)	No	Local critically ill. ICU patients in Massachesets teaching hospitals	Ghassemi et al. [127]
	National Prescription Audit (NPA) and National Disease and Therapeutic Index (NDTI)	No	National sample. Commercial medication-focused databases from IMS Institute for Healthcare Informatics	Alexander et al. [128]
	New York Presbytarian	No	Local. Single hospital. 30 years+ of data	Melamed et al. [129]
	Palo Alto Medical Foundation (PAMF)	No	Regional. Single HMO. HMO network member	Goyal et al. [130]
	Partners Healthcare	No	Regional. Single HMO. Feeds into i2b2	Castro et al. [131]
	PharMetrics Patient-Centric Database, now merged with IMS databases	No	National sample. Pharmacy and encounter data 14 m people	Berger et al. [132]
	Penn Longitudinal Database	Yes	Regional. Public mental health use (secondary care) in Philadelphia. Also part of collaborative perinatal project	Connolly Gibbons et al. [133]
	Shared Health Information Network (SHRINE)	No	Multiple sites. Secondary care. Collaboration between Harvard and University of California hospitals	Kohane [134]
	Scalable Partnering Network (SPAN) for Comparative Effectiveness Research (CER)	No	National sample. Project providing linkage between nine HMOs and two community partners	Toh et al. [38]
	Stanford Translational Research Integrated Database Environment (STRIDE)	No	Local. Data from healthcare provider. Data on 2 m people since 1994	Raj et al. [135]
	Texas Department of Criminal Justice	No	Local database of prisoners	Baillargeon et al. [136]
	University of Michigan Health System data warehouse	No	Local secondary healthcare provider. Uses Electronic Medical Record Search Engine (EMERSE)	Hanauer et al. [137]
	Vanderbilt University Biorepository—BioVU	No	Local secondary care provider. Genomics, select health metrics and EHR	Crawford et al. [138]
	Veterans Affairs Database	No	National specialist provider for veterans. Provides healthcare for aprox 14 m, has smaller biobank	Bauer et al. [139]
Multi-continent				
	Aetionomy	Yes	Neurodegenerative diseases. Under European Innovative Medicines Initiative, aligned with EPAD in Europe and GAP in North America	Hofmann-Apitius et al. [140]
	Asian Pharmacoepidemiology Network (AsPEN)	No	Eight cohorts in distributed network model: six countries, four continents, 200 m people	Pratt et al. [141]
	Enhancing NeuroImaging Genetics through Meta-Analysis (ENIGMA)	Yes	Sets of research cohorts. 70 institutions taking part	Thompson et al. [60]
	Global Burden of Disease (GBD)/WHO mental health survey	No	Estimates of morbidity for 187 countries	Whiteford et al. [57]
	Genetic Consortium for Anorexia Nervosa	Yes	Up to 30 datasets for GWAS	Reichborn-Kjennerud et al. [142]
	Health Care Quality Indicators (HCQI) for OECD countries	No	Comparative data on national health systems	Moran and Jacobs[143]
	IMS Prescribing Insights database	No	Medication-based database. Presence in 30 countries	Wong et al. [144]
	Psychiatric Genomic Consortium	Yes	Has a number of working groups for specific disorders and cross-disorder group	Cross-Disorder Group of the Psychiatric Genomics [59]
	International Genomics of Alzheimer’s Project (I-GAP)	Yes	Including existing genetic consortia and other cohorts	Lambert et al. [145]
	Sequenced Treatment Alternatives to Relieve Depression (STAR*D)	Yes	Whilst this international study was not “big data”, in terms of using hybrid EHR and manual methods, it develops techniques to be used for observational research in big data	Garriock et al. [146]
	WHO Global Health Observatory Data Repository	No	Special topics covered, including mental health and suicide	WHO [147]

**Fig. 1:**
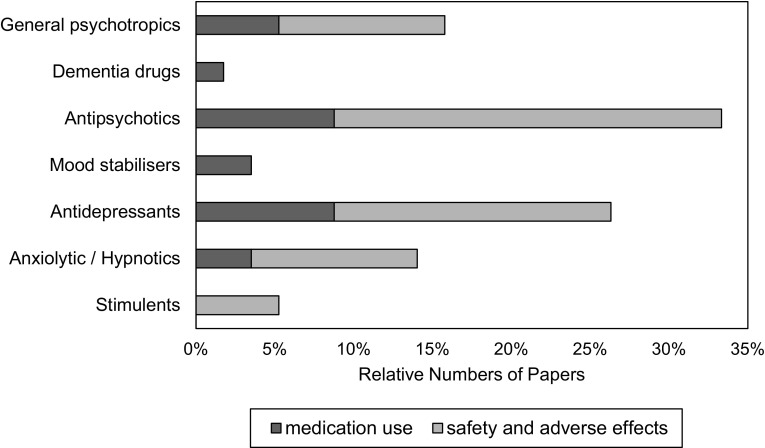
The relative number of papers found reporting on different classes of medication (57 papers on medication in total)

Distributions of identified reports by disorder and nature of research are summarised in Table 2 with examples, although it is important to bear in mind that percentages refer to studies identified in this review which will not have been exhaustive; they are included for illustrative purposes and inferences regarding the total literature should be appropriately circumspect. The disorders covered in the papers we identified show that big data resources had been used most commonly to research unipolar depression and dementia, followed by schizophreniform and autism spectrum disorders, and relatively uncommon output on bipolar disorder, substance use disorders and neurodevelopmental disorders. For most disorders, the output was reasonably equally split between epidemiological/aetiological research and analyses of treatments and outcomes. The distributions of medication-reporting publications are summarised in Fig. 1 and indicate a predominant focus on antipsychotic and antidepressant agents, with relatively few publications on mood stabilisers or treatments for dementia. Specific examples of papers on medication and other topics are given in Table 3. Beyond medication profiles and safety, there were a number of papers on suicide, service use and user characteristics. Few of the research studies that we found were directly focused on mental health policy, but their findings often have important policy implications. A more detailed narrative of the types of questions addressed forms the focus for discussion of the topic.

**Table 2:** Example topics in papers discussing mental illness epidemiology, treatment and outcome

Disorder (% of papers)	Descriptive epidemiology and service use	Risk factors, comorbidities and genetics	Treatment and prognosis	Physical health, pregnancy, mortality
All disorders (10 %)	Manson [148] Heggestadet al. [149]	Roque et al. [150] Cross-Disorder Group of the Psychiatric Genomics [59]	Donisi et al. [89]	Perini et al. [151] Lawrence et al. [82]
Severe mental illness (5 %)	Lyalina et al. [152]	Kyaga et al. [153] Steinberg et al. [154]	Perlman et al. [112]	Matheson et al. [155] Wangel et al. [156]
Dementia (9 %)	Knopman et al. [24] Kosteniuk et al. [157]	Exalto et al. [158] Lambert et al. [145]	van den Bussche et al. [159]	Rait et al. [160]
Substance use disorder (2 %)	Bonn-Miller et al. [161]	Nesvåg [162]	Mark et al. [163]	
Schizophrenia (6 %)	Okkels et al. [164] Evensen et al. [102]	Harper et al. [165]	Stroup et al. [166]	Gal et al. [167] Vigod et al. [168]
Bipolar disorder (2 %)	Castro et al. [169]	Schaefer et al. [170]	Hayes et al. [171]	Lee and Lin [172]
Depressive disorders (11 %)	Hoffmann et al. [173] Wong et al. [35]	Hanauer et al. [137] Ul-Haq et al. [174]	Morkem et al. [34] Musliner et al. [175]	Lin et al. [119]
Anxiety and somatoform disorders (2 %)	Walters et al. [75]	Lacourt et al. [176]	Sandelin et al. [177]	Frayne et al. [178]
Eating disorders (1 %)	Micali et al. [179]	Reichborn-Kjennerudet al. [142]		
Post-partum mental disorders (2 %)	Polachek et al. [180]	Goyal et al. [130]		
Intellectual disabilities (1 %)		Sprung et al. [181]		Alexander et al. [182]
Autism/Autism Spectrum Disorder (ASD) (6 %)	Kohane [134]	Hsu et al. [183] Clarke et al. [184]	Wong et al. [144] Murphy and Spooren [107]	
Other neuro-developmental disorders (4 %)	Surén et al. [185]	Leivonen et al. [186]	Hoffmannet al. [187]

**Table 3:** Examples of other topics appearing in multiple papers

Topic (% of papers)	Example papers
Medication prescription 6 %	Sultana et al. [86] John et al. [95] Abdullah-Koolmees et al. [188] Hartz et al. [189]
Medication safety and adverse drug reactions 13 %	Chung et al. [190] Eriksson et al. [191] Castro et al. [131]
Medication safety in older adults	Hwang et al. [111] Huybrechts et al. [54]
Medication safety during pregnancy	Hviid et al. [192] Palmsten et al. [193]
Suicide and self-injury 5 %	Stewart et al. [194] Simon et al. [195]
Mental health admissions 4 %	Frick et al. [88] Bardach et al. [196]
Patient characteristics 4 %	Koopmans et al. [197] Oram et al. [198]
Mental Health Services Quality 3 %	Moran and Jacobs [143]

## Discussion

A wide variety of big data resources are emerging as platforms for mental health research, and it is inevitable that the characteristics of these resources will shape the questions addressed, particularly data availability. At one end, there are databases that take full clinical data directly from the electronic health record (EHR) at primary care or hospital level; some databases are populated from specific patient-level information provided by health service staff for the process of research or surveillance; some make secondary use of unmodified administrative data; some rely on patient report. Some studies transcend boundaries by making use of massed service-level data—such as the European Observatory of Health Systems and Policies—or combine findings from different databases—such as the Psychiatric Genomics Consortium. We have sought in this review to provide a snapshot of big data resources which are now becoming available for clinical/epidemiological mental health research and the way in which these have been used to date. It would be difficult to guarantee comprehensiveness in coverage due to limitations in our search methodology, the fast pace of current development in this field, the under-acknowledgement of the role of databases, and the nature of much of the research (i.e. not published in peer-review/indexed journals). In addition, the data resources themselves do not exist within tightly definable boundaries. For example: general healthcare databases may contain mental health relevant information but may not have been used for research within this field; many biological databases might be classifiable as ‘big data’ because of the density of information contained; and there is no clear point at which information from a large survey, or series of surveys, or cohort study, becomes large and detailed enough to be called ‘big data’. We have referenced resources that have access to large numbers of individuals, and have sought to provide examples that are broadly representative of emerging information available. For example, we have cited administrative data registries with linked death certification records to investigate mortality in mental disorders, and we have described these as big data; however, there is no qualitative difference between this and the linkage of the large Norwegian HUNT survey of over 60,000 community residents to national data on mortality and occupation-related outcomes [11, 12], which tends to be described instead as a large cohort study rather than ‘big data’. Similarly, this review did not attempt to cover large cohort studies with an emphasis on original data collection rather than reliant on administrative data (e.g. in a UK context, cohorts such as ALSPAC, Whitehall, or the 1946, 1957 or 1970 birth cohorts)—whose boundaries with big data are inevitably indistinct. Big data resources, thus, tend to be defined by the challenges faced by the data and their interpretation, as will now be described, rather than solely by the size or complexity of a database.

### Big data and the five V’s

Big data resources are often characterised by ‘Vs’: originally three (volume, velocity and variety), now five (adding variability and veracity), but with the potential for further expansion (e.g. visualisation and value: http://dataconomy.com/seven-vs-big-data/). Taking the five V’s as the most common current characterisation, it is worthwhile considering each in turn as it applies to the mental health relevant databases described here:

1. The examples we identified exemplify ‘volume’ in the large number of cases represented and, in many instances, the quantity of information on each person represented. This particularly applies to healthcare data which are linked to high-compute biological datasets (e.g. from ‘omics’ and imaging) and to those which include the full electronic health record—i.e. which contain both large case numbers and large amounts of detail on each case. While small compared to many ‘big data’ resources, electronic health records represent a step-change in volume compared to the administrative databases previously relied on for analysis.
2. ‘Velocity’ may be a feature of electronic health records databases if these accumulate in real time, although is less relevant to static and/or periodically updated sets, and depends on the way in which a database is used. At the moment most research use has been observational, using historic data extractions and therefore not encountering the velocity challenge, even in ‘live’ (i.e. continually accumulating) databases. This will change once interventions start being developed which rely on real-time data feeds from health records, and will be challenge not only for hardware (e.g. the demands on central or local processing hubs) but also for designing appropriately agile software to enable such processing.
3. ‘Variety’ has also been less relevant to date because most analyses are still focusing on relatively stereotyped datasets drawn from original or derived structured fields; however, this is changing with increasing interest in natural language processing to derive information from text—whether relatively simple information extraction applications to render pre-defined constructs available as structured fields, or more complex whole-text analytics (e.g. investigating subtle changes in health records text as a potential predictor of adverse events such as suicidal behaviour https://slamtwigops.wordpress.com/tag/e-host-it/). ‘Variety’ will also become an increasingly relevant consideration as health records databases begin to integrate with the large-volume information generated by devices and remote monitoring, as well as potentially from patient-entered data—or example, when considering the differences in wording used to describe the experience of a disorder between a clinician writing in the health record and someone with the condition contributing to an online forum.
4. ‘Variability’ is used to describe the phenomenon of data whose meaning is constantly changing. Within health records, data fields clearly do change over time in the way information is entered, although this is generally at a pace which is manageable. Text fields in health records may present more of a challenge, as there are likely to be more rapid and less manageable changes in the ways clinicians record information, although this is likely to be negligible compared to the rapid evolution in social media and the language used there (and thus in any development of shared records with the facility for accommodating patient-entered information).
5. ‘Veracity’ is perhaps the most important challenge in the use of any administrative database for research, simply because source data have not generally been collected with research in mind and thus it is important to be aware of factors influencing the recording of information or not, and the accuracy with which this is carried out. The veracity challenge will be considered later in this discussion, having first reviewed the data resources available.

### Electronic health records

EHRs present novel opportunities for research because of the very large volumes of information which naturally accrue and, unlike paper-based records, are accessible without prohibitively time-consuming data entry. Considering volume of information, there is a major distinction between databases using only structured fields, and those using the free text [13, 14]. Structured data such as age, sex, diagnosis, and dates of service-level events (admissions, discharges, etc.) are routinely entered by clinical or administrative staff, can be made readily available for research use, and are relatively easily de-identified for data governance requirements. However, the fact that structured information is more readily available for analysis does not make it any more valid or accurate than unstructured information. Clinical uncertainties can be poorly translated into codes [15–17], and the sustainability of imposed structured data entry in routine clinical care (e.g. through embedded checklists and scales in the EHR) remains to be established. Free text is typically extensive in case note fields and uploaded correspondence for mental health EHRs, but less accessible for analysis, and less easily anonymised; however, text-contained information is potentially the most valuable for research despite the inconvenience of having to design mechanisms for extracting the information.

To make better use of the whole record, text mining tools have attracted increasing interest as a means of facilitating research with free text alongside the structured record [18–21]. This can increase sensitivity for record identification; for example, Vanderbilt University Medical Centre found that extraction of diagnosis of dementia from structured fields identified 38 % of cases found by manual notes review, whereas 91 % of these were identified through a free text information extraction application [22]. However, it should be noted that even searching the free text for a diagnosis will only give an accurate indication of the numbers of people identified with a disorder, which may be a substantial underestimate of community cases. For example, Mayo Clinic analyses found that, of people identified in research studies as having definite dementia or autism spectrum disorder, around 70 and 50 %, respectively, had any note of such in their EHR [23, 24].

A key potential advantage of using information derived from EHR free text is the quantity of phenotypic data beyond a diagnosis, both in terms of patients’ mental health—such as symptom profile [25] or treatment responsiveness [26]—and the context in which a disorder is occurring [27]. This can be used for highlighting patients who have inclusion criteria for recruitment into observational or interventional studies, or can be used to investigate treatment response directly within the database: all relevant for the development of personalised medicine [28, 29]. Furthermore, phenotypic signatures of direct clinical relevance, such as “high suicide risk” or “vulnerable to depression”, might be fed back in real time via the EHR to alert the treating clinician [30, 31], coupled with decision support software or information resources. Free text can also be mined to define groups or outcomes that are too rare to be studied conventionally—such as the use of Khat in South-East London [32] or neuroleptic malignant syndrome [33].

Primary Care EHRs are potentially valuable for investigating the wider health of people with common or severe mental illnesses. Examples include Canadian longitudinal research into changes in the diagnosis and treatment of depression [34, 35], or the use of a UK general practice database to investigate the risk of cardiovascular disease in people with severe mental illness and to derive a risk prediction model for this outcome [36]. While some countries benefit from large healthcare providers with associated data resources (e.g. National Health Service data in the UK, and the Taiwan National Health Insurance Research Database), others, such as the USA, have brought together healthcare providers in ‘virtual networks’ [37, 38]. Anonymised data derived from each provider’s EHRs can be brought together with tools such as the Health Care Systems Research Network’s online integration tool “PopMedNet” for research, or to compare practices, such as the benchmarking of psychotropic prescribing [39]. There are also EHR-genomic consortia, such as eMerge—a collaboration of Marshfield, Mayo Clinic, Northwestern, Group Health and Vanderbilt—which hosts some dementia cohorts [40].

It is important that the governance of these EHR databases and projects is planned to balance the concerns of patients and the needs of researchers. Full anonymization may not be possible for projects requiring phenotypic details [41]; other protections such as limited access and firewalls must therefore be considered so as not to lose “social licence” for these types of projects [42–44]. Both researchers and patients should have input to the next generation of data repositories and projects to shape them towards the kinds of questions that remain outstanding, such as capturing traits as well as diseases for research compatible with the USA’s National Institute of Mental Health Research Domain Criteria (RDoC) paradigm [45, 46].

### Case registers involving de novo data collection

Specialist databases form registries of people in contact with the mental health system, or have evolved from this to offer surveillance of both service users and the services themselves [6]. While it is possible to create and maintain such a register solely with electronic health records, many involve the collection of specific data, usually requested from the service providers. These databases are a helpful resource for research into patterns of service use and their individual and societal determinants. Some databases, such as the Mental Health National Outcomes and Casemix Collection in Australia and the DGPPN-BADO in Germany have made efforts to include valid measures of outcome for service users, which helps them monitor improvement [47, 48] and also for research, such as into treatments for depression in Germany. There are also examples of more specialised registries: a database in the Netherlands recording seclusion and restraint episodes looking for insights to drive service improvement [49]; and the GRiST mental health data set in the UK, which deals with risk assessment and aims to use the data to become a decision support tool [50, 51].

### Administrative databases

We identified a number of examples of projects making secondary use of large-volume administrative data to draw conclusions about healthcare use through diagnoses on hospital discharge notifications, billing for procedures, or prescriptions. Some of these databases are long established, such as the Swedish population-based registers; while the expansion of Medicaid, and the requirement for billing with ICD-codes, combined with incentives for “meaningful use” of information technology [52], has led to large accumulations of new data resources. This information tends to be used to describe treated prevalences of disorders, patterns of prescribing, and comparisons of ‘real-world’ treatment with recommendations. Where data include both prescriptions and incident diagnosis, this can also be useful in pharmacovigilance, using retrospective cohort or nested case–control studies to investigate adverse events [53], such as differences in the safety of different antipsychotics in older patients examined using Medicaid billing data for nursing home residents [54]. Such data can also be used to describe treatment costs—which may have a specific focus, such as a US investigation of the cost of non-compliance in bipolar disorder [55], or a broader scope, such as an EU project investigating whether the financing of health services in different EU countries affects the quality of mental healthcare [56].

### Surveys and biobanks

In most circumstances, surveys and interviews are not practical for assembling big data resources; however, the WHO Global Burden of Disease programme uses standardised mental health surveys (based on the Composite Diagnostic Interview—CIDI) carried out at intervals by local research teams in member countries [57], and could be claimed to come closest to being a ‘big data’ survey in the mental health field. Large samples have also been achieved by some biobanks, such as UK Biobank, which already has self-report data for 500,000 [58] and is looking to improve its mental health phenotyping through an online questionnaire based on the CIDI-short form. Genome Wide Association Studies (GWAS) for complex disorders require large independent datasets of genomes, therefore it makes sense for researchers on projects such as UK Biobank to co-operate with others. The international Psychiatric Genomics Consortium (PGC) is a means to achieve this, sharing both datasets and expertise [59]. International research collaborations have also allowed the leveraging of neuroimaging taking place in different locations through the ENIGMA program [60].

### Record linkage

All of the above databases can be given new dimensions when data from other sources are linked at the level of the individual [3, 9]. This is facilitated in countries with a unique identification number for its residents, such as many of the Nordic countries: for example, allowing researchers using Sweden’s population-based registers to link reports of death by suicide to records of psychiatric and medical diagnoses, periods of sickness absence from work, and unemployment [61–65]. Danish records that link also to parents and siblings have been used to investigate potential risk factors for schizophrenia, such as family history, season of birth, urban living, and trauma to mother during pregnancy [66–68]. A number of observational studies have investigated the safety of psychotropic medication in pregnancy, but in Western Australia this approach has been taken one step further by assembling and following an e-cohort of children born to mothers who have schizophrenia, using health and social service administrative registers alone; associations with obstetric complications and subsequent intellectual disability have already been reported [69, 70].

### Data veracity

Although it might be assumed that cohorts assembled through researcher interviews are preferable to those derived from administrative data, it is important to recognise that each research method has strengths and limitations. Research interviews do provide potentially highly accurate information about a person’s status at a particular time; however, conventional research projects are limited, and not just in the numbers of cases who can be interviewed and examined. They may also poorly capture variability or trajectories in health status over time (especially as recollection of episodes of mental disorder has been found to be so poor [71]) which may be better characterised from administrative data than retrospective interview. Furthermore, even highly trained interviewers might have difficulty ascertaining phenomena like physical signs or relatively rare symptoms, which may be better identified from clinician-derived text in health records.

Conversely, as previously discussed, a veracity challenge for all healthcare databases is that information used has not, generally, been collected for research reasons; therefore, data are vulnerable to influence from forces other than the underlying patterns of disease, and hence the incentives for record-keeping need to be taken into account (sometimes considered under a ‘data provenance’ heading). One important issue concerns diagnosis, as many studies rely on recorded diagnostic information and frequently do not have any further information on the disorder under investigation beyond this. However, many mental disorders do not result in a documented diagnosis because the person does not report the disorder to a healthcare practitioner, because the practitioner does not identify the disorder, or because they do not assign or record a diagnosis. For example, in 2009 it was estimated that one-third of all people living in England with dementia had received a formal diagnosis [72]. Since then, political pressure, availability of medication and other factors have changed the culture surrounding the making and recording of a dementia diagnosis. Registers of people with dementia kept by all GPs in primary care have consequently been increasing in size by an average of 8 % per year [73]—a change that does not reflect changing epidemiology of the disease. Primary Care diagnosis rates of anxiety and depression in the UK have also been found not to be representative of disease trends [74, 75]; however, a team at the Secure Anonymised Information Linkage Databank in Wales found that combining diagnosis and symptom terms appeared to be more reflective, which suggests the wisdom of working with people who are familiar with the data source being used [76].

## Conclusion

Big data are being used for mental health research in many parts of the world, and for many different purposes. Secondary use of administrative data, especially where routine diagnostic information is included, is likely to become increasingly adopted for research as these information resources are relatively inexpensive and scalable. Furthermore, secondary use of clinical information is coming up fast behind. ‘Volume’ challenges can generally be addressed with current information storage capacity and availability. The ‘velocity’ challenge is yet to be addressed because most of these big data resources are static and updated periodically, with few ‘real-time’ applications currently developed; however, this situation will change if decision support applications are implemented, and/or if learning and artificial intelligence begin to be incorporated in records systems. ‘Variety’ and ‘variability’, like velocity, are not current challenges but are likely to become increasingly salient in the near future. ‘Veracity’ remains a key consideration and one which is unlikely to change with technological advances, because secondary data use continues to depend on the data actually being recorded in clinical practice. The other big considerations are data governance and security, which clearly require robust planning and an effective, ongoing public dialogue.

An over-arching conclusion from this review is that research questions continue to be shaped by the information that happens to be available and accessible in these data resources. For example, the fact that healthcare databases are used so extensively for medication-oriented research questions is likely to reflect the relative ease with which medication data can be extracted. Equally their lack of use for investigations of symptom profiles or illicit substance use reflects the lack of structured data on these constructs in most records systems. A transition is likely to be needed whereby the data resources themselves are shaped, at least to some extent, by research priorities; however, this is only likely to be effective if the research priorities, in turn, are shaped by the needs of clinical services and those who use them.
